# Antimicrobial Constituents of *Artemisia afra* Jacq. ex Willd. against Periodontal Pathogens

**DOI:** 10.1155/2012/252758

**Published:** 2012-05-30

**Authors:** Garland More, Namrita Lall, Ahmed Hussein, Thilivhali Emmanuel Tshikalange

**Affiliations:** ^1^Department of Plant Science, University of Pretoria, Pretoria 0002, South Africa; ^2^Chemitsry Deparment, Universirty of Western Cape, Private Bag X17, Bellvile 7535, South Africa

## Abstract

The phytochemical investigation of an ethanol extract of *Artemisia afra* led to the isolation of six known compounds, acacetin **(1)**, 12**α**,4**α**-dihydroxybishopsolicepolide **(2)**, scopoletin **(3)**, **α**-amyrin **(4)**, phytol **(5)**, and a pentacyclic triterpenoid betulinic acid **(6)**. The compounds were evaluated for antimicrobial activity against Gram positive (*Actinomyces naeslundii, Actinomyces israelii, * and *Streptococcus mutans*), Gram negative bacteria (*Prevotella intermedia, Porphyromonas gingivalis and Aggregatibacter actinomycetemcomitans* previously known as *Actinobacillus actinomycetemcomitans*), and *Candida albicans*. The crude extract of *A. afra* inhibited the growth of all tested microbial species at concentration range of 1.6 mg/mL to 25 mg/mL. The compounds 1–6 also showed activity range at 1.0 mg/mL to 0.25 mg/mL. Three best compounds (scopoletin, betulinic acid, and acacetin) which showed good antimicrobial activity were selected for further studies. Cytotoxicity of extract and compounds was determined using the XTT cell proliferation kit. The antioxidant activity of the extract and compounds was done using the DPPH scavenging method. The extract showed good antioxidant activity with an IC_50_ value of 22.2 **μ**g/mL. Scopoletin had a strong transformation of the DPPH radical into its reduced form, with an IC_50_ value of 1.24 **μ**g/mL which was significant to that of vitamin C (1.22 **μ**g/mL). Acacetin and betulinic acid exhibited a decreased scavenging activity with the IC_50_ of 2.39 and 2.42 **μ**g/mL, respectively. The extract and compounds showed moderate toxicity on McCoy fibroblast cell line and scopoletin was relatively nontoxic with an IC_50_ value of 132.5 **μ**g/mL. Acacetin and betulinic acid also showed a smooth trend of non-toxic effects with IC_50_ values of 35.44 and 30.96 **μ**g/mL. The obtained results in this study confirm the use of *A. afra* in the treatment of microbial infections.

## 1. Introduction

Periodontal disease is a chronic, multifactorial disease of the tissue supporting teeth [1]. It is characterised by local infection and inflammation in teeth supporting tissue leading to connective tissue destruction and alveolar bone loss [2]. The etiology of periodontitis is the oral bacteria [3, 4]. If left untreated, periodontitis can have medical consequences such as weight loss, chronic pain, sore or loss teeth, swollen gums, tooth decay, breakage of the maxillary or mandibular bones, and renal, coronary, and hepatic diseases [5–7]. The population of periodontal bacteria begins to increase as an anaerobic environment is produced due to oxygen scavenging activity of the early subgingival colonizers [4]. These bacterial populations lead to biofilm formation which consists of microcolonies, extracellular layers, fluid channels, and communication systems [8]. Biofilms may consist of more than 700 different microbial species which leave symbiotically and incubate each other [9]. The biofilm formation and associated disease can be prevented by daily tooth brushing and chemotherapeutic agents such as chlorhexidine (CHX), fluorides biguanide antiseptics, quaternary ammonium-antiseptics and phenol derivatives [10, 11]. Although these chemotherapeutic agents are effective they can cause side effects, such as gastrointestinal irritation, tooth staining, and gum irritation [11].

Dental treatment usually is expensive and not so easily accessible, especially in developing countries; therefore humans have turned to the use of natural traditional remedies to prevent oral ailments [12–15]. *Artemisia afra* (African wormwood, family Asteraceae) is widely distributed along the eastern parts of Africa. It grows in thick, bushy areas, usually with tall stems up to 2 m high but sometimes as low as 0.6 m. *A. afra *is a common species in South Africa with a wide distribution from the Cederberg Mountains in the Cape, northwards to tropical East Africa and stretching as far north as Ethiopia [16, 17]. Worldwide there are about 500 species of *Artemisia*, mainly from the northern hemisphere. Many of the other *Artemisia* species are aromatic perennials and are used medicinally [18]. In southern Africa it is used to treat coughs, colds, diabetes, malaria, sore throat, asthma, headache, dental care, gout and intestinal worms [19]. *In vitro* studies done have revealed that* A. afra* is a potential antidepressant, cardiovascular, spasmolytic effects, antioxidant, and antimycobacteria [18, 20, 21]. Furthermore, the extracts of this plant species have shown activity against *Trypanosoma brucei brucei* [22].

 The rationale of this study was to determine the antimicrobial, antioxidant, and cytotoxicity of *Artemisia afra* and isolated compounds against oral microorganisms which are responsible for dental caries, gingivitis, and periodontitis.

## 2. Materials and Methods 

### 2.1. Plant Material

*Artemisia afra* was collected at the South African National Botanical Institute (SANBI), Pretoria. Voucher specimen was prepared and identified at the H. G. W. J. Schwelcherdt Herbarium (PRU), University of Pretoria.

### 2.2. Preparation of Extracts

Fresh plant material was soaked in 96% ethanol and homogenized into fine mesh. The extract was then filtered through the Whatman No.1 filter paper. The filtrates were evaporated to dryness in a (BUCHI) Rotavapor under reduced pressure of 40°C.

### 2.3. Antimicrobial Assay

#### 2.3.1. Microbial Strains

The microorganisms used in this study include *Actinomyces naeslundii *(ATCC 19039),* Actinomyces israelii *(ATCC 10049),* Aggregatibacter actinomycetemcomitans *(ATCC 33384),* Candida albicans *(Med I),* Porphyromonas gingivalis *(ATCC 33277),* Prevotella intermedia* (ATCC 25611),* and Streptococcus mutans *(ATCC 25175). Bacteria were grown in the Casein-peptone Soy Agar medium (CASO) (Merck SA (Pty) Ltd.) under anaerobic conditions in a jar with anaerocult A (Merck SA (Pty) Ltd.), at 37°C for 48 hours. Sabouraud Dextrose Agar medium (SDA) (Merck SA (Pty) Ltd.) was used for the culturing of *Candida albicans* and incubated at 37°C for 24 hours under aerobic conditions. Subculturing was done once weekly.

### 2.4. Determination of Minimum Inhibitory Concentration (MIC) and Minimum Microbicidal Concentration (MMC) 

The microdilution technique using 96-well microplates [23] was used to obtain the MIC and MMC values of the crude extract against microorganisms under study. The extract was serially diluted in the 96-well plate with 48 hours old microorganisms (5 × 10^6^ CFU/mL) grown at 37°C and the final concentration of extract and positive control (CHX) ranged from 25.0 mg/mL to 0.8 mg/mL. Microbial growth was indicated by adding 40 *μ*L of (0.2 mg/mL) *p*-iodonitrotetrazolium violet (INT) (Sigma-Aldrich, South Africa) to microplate wells and incubated at 37°C for 48 hours. MIC was defined as the lowest concentration that inhibited the colour change of INT. The MMC was determined by adding 50 *μ*L of the suspensions from the wells, which did not show any growth after incubation during MIC assays, to 150 *μ*L of fresh broth. These suspensions were reincubated at 37°C for 48 hours. The MMC was determined as the lowest concentration of extract which inhibited 100% growth of microorganisms [24].

### 2.5. Antioxidant Activity

The free radical scavenging activity was measured using 1, 1 diphenyl-2-picryl-hydraxyl (DPPH) assay [25] with slight modifications. The ethanol extract of* A. afra *and Vitamin C (positive control) 1000 *μ*g/mL (20 *μ*L) was added in the first three wells of a 96-well plate containing 200 *μ*L of distilled water to make up final concentration of 100 *μ*g/mL and the remaining wells were filled with 110 *μ*L of distilled water. The first raws containing the extract/compounds were serially diluted to wells which contain 110 *μ*L of distilled water, and later, 90 *μ*L of methanolic solution of DPPH (90 mM) was added to all the wells. The final concentrations of the extract/compounds ranged from 100 to 0.8 *μ*g/mL. The plates were incubated at 37°C for 30 min and the absorbance was measured at 517 nm using the ELISA plate reader. The percent radical scavenging activity by *A. afra* was determined by comparison with ethanol (blank). The inhibition ratio was calculated as follows: % DPPH radical-scavenging = (AC-AS)/AC × 100, where AC is absorbance of the control solution (containing only DPPH solution), and AS is the absorbance of the sample in DPPH solution. The percentage of DPPH radical-scavenging was plotted against the plant extract/compounds concentrations (*μ*g/mL) to determine the concentration of extract/compound required to scavenge DPPH by 50% (EC_50_).

### 2.6. Determination of Cytotoxicity

#### 2.6.1. Preparation of Extract and Compounds

Extract and compounds were dissolved in dimethyl sulfoxide (DMSO) and stored at −20°C. All tested compounds were diluted to the final concentration with RPMI 1640 and control cultures were diluted with 0.1% DMSO.

#### 2.6.2. Cell Culture

McCoy cells were maintained in monolayer culture at 37°C and 5% CO_2 _ with 10% PBS Medium, 10 *μ*g/mL of penicillin, 10 *μ*g/mL, streptomycin, 40 *μ*g/mL gentamycin, and 0.25 *μ*g/mL fungizone.

#### 2.6.3. Cell Proliferation Assay

A microtiter plate with McCoy cells was used for testing all the ethanol extracts for cytotoxicity following the method of [26]. Cytotoxicity was measured by the XTT (sodium 3′-[1-(phenyl amino-carbonyl)-3,4-tetrazolium]-bis-[4-methoxy-6-nitro] benzene sulfonic acid hydrate) method using a cell proliferation kit II (Roche Diagnostics GmbH). Hundred microlitres of McCoy cells (1 × 10^5^ mL) was seeded onto a microtiter plate and incubated for 24 h to allow the cells to attach to the bottom of the plate. Dilution series were made of the extract and compound and the various concentrations (400 to 3.1 *μ*g/mL) were added to the microtitre plate and incubated for 48 h. The XTT reagents were added to a final concentration of 0.3 mg/mL and the cells were incubated for 1-2 hours. The positive drug control (Zearalenone) at concentrations range of (10 *μ*g/mL to 0.6 *μ*g/mL) was included in the assay. After incubation the absorbance of the colour was spectrophotometrically quantified using an ELISA plate reader, which measured the optical density at 490 nm with a reference wavelength of 690 nm. The assay was carried out in triplicate.

#### 2.6.4. Statistical Analysis

Statistical analysis was conveyed as means ± SD using GraphPad Prism 4.0 with a significant difference of (*P* < 0.05).

## 3. Isolation and Identification of Compounds

The antibacterial compounds present in *A. afra* extract were determined by the direct bioautography method of chromatograms using *S*.* mutans* [27]. The extract was spotted onto a TLC plate and developed using hexane: ethyl acetate at different ratios (1 : 1, 3 : 7, and 7 : 3). The plates were thoroughly dried and then the chromatograms were sprayed with a dense culture of *S. mutans*, incubated overnight at 37°C. The plates were further sprayed with 0.2 mg/mL of *p*-iodonitrotetrazolium (INT) (Sigma). Clear zones of inhibition indicated compounds which inhibited bacterial growth. The isolation of compounds was performed using column chromatography. Fractionation was preceded by using silica gel 60 (70–230 mesh) and Sephadex LH 20. Thin layer chromatography (TLC) was performed on aluminum sheets coated with silica gel 60 F_254 _ (Merck) and UV light was used to detect compounds. TLC plates were further sprayed with vanillin/sulphuric acid reagent. H^1^-NMR and ^13^C-NMR spectra were obtained by using Nuclear magnetic resonance (NMR) Germin 200 AT 199, 50 Hz, respectively.

The Isolation of *A. afra* was started with 200 g of ethanol extract on to a 100 mm diameter column. The column was filled with 2 kg silica gel, eluted with a mixture of hexane: ethyl acetate of increasing polarity (100 : 0 to 0 : 100). Forty fractions were obtained and combined to make up 12 (I-XII) main fractions according to similarities of compounds as determined by TLC plate. A Sephadex column was conducted on the fraction X using 100% MeOH and it yielded 25 subfractions which were combined in to three subfractions (L, M, N). Fraction N was fractionated using MeOH and yielded a pure compound **1**. Fraction M was isolated using a gradient of solvents DCM: Hex, 9 : 1 increasing polarity to 3% to obtain a florescent blue compound **3**.

Fraction VII was chromatographed using DCM: MeOH (95 : 5), and 20 fractions were obtained and combined to 3 subfractions. Fraction G was fractionated using DCM: MeOH, increasing polarity on a Sephadex column and two fractions were obtained of which one was a pure compound **2**, the second fraction was again fractionated using DCM: MeOH (95 : 5) and compound **6 **was obtained. Compound **5 **was isolated using Hex: EtoAc, at a ratio of 9 : 1, using silica gel column. A succession of a blue-coloured compound was observed on a TLC plate after application of vanillin/sulphuric acid. Subfraction H from fraction VII was chromatographed using a sephadex column with ethanol as its mobile phase and it yielded a white precipitate of a pure compound **4**.

## 4. Results and Discussion

**Figure 1:**
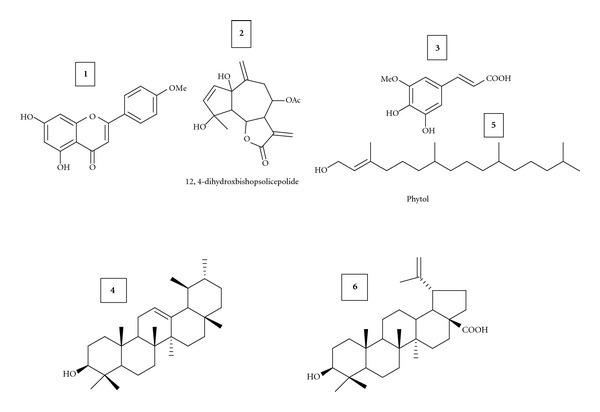
Chemical structures of compounds isolated from the aerial part of *Artemisia afra. *

The TLC profile gave a clear antibacterial activity of the extract and guide to isolate ideal compounds. In all solvent systems tested on TLC, both polar and nonpolar bends demonstrated activity by inhibiting the growth of *S. mutans*. Isolation from *A. afra* extract yielded six compounds (Figure 1), one known flavone Acacetin **(1)**, sequiterpene12*α*,4*α*-dihydroxybishopsolicepolide **(2)**, a diterpene Phytol **(5) **[28, 29], and two pentacyclic triterpenes **(4)**. A thorough revision of literature indicated that the data for pentacyclic triterpene matched with those of *α*-Amyrin, a widely spread triterpene in nature [30, 31]. The presence of the signal at 3.0 of H-17 confirms the structure of Betulinic acid **(6)**. We have compound **(3)** of which the forgoing data is identical with the known compound Scopoletin. All these compounds were characterized by their ^1^H-NMR and ^13^C-NMR spectra.

The strong antimicrobial activity demonstrated by the ethanol extract of* A. afra* has provided us with more evidence that needed further chemical investigation of bioactive compounds present. The extract of *A. afra* showed good inhibitory effects against all Gram positive bacteria. It can be noted that the MIC and MMC values varied from 1.6 to 25.0 mg/mL among Gram positive bacteria. *C. albicans* which is a thick grower fungus was inhibited at a concentration of 6.3 mg/mL. However, the MMC value insignificant as compared to the results of the MIC. The *A. actinomycetemcomitans* was the resistant Gram negative bacteria as compared to all other tested bacteria (Table 1). The lowest MIC and MMC value of isolated compounds was recorded at 0.25 mg/mL of compounds **(6)**, **(3)**, and **(1)** against *A. israelii *and *A. naeslundii.* Of all microorganisms, *A. actinomycetemcomitans *and* C. albicans *showed to be resistant against all compounds tested (Table 1).

**Figure 2:**
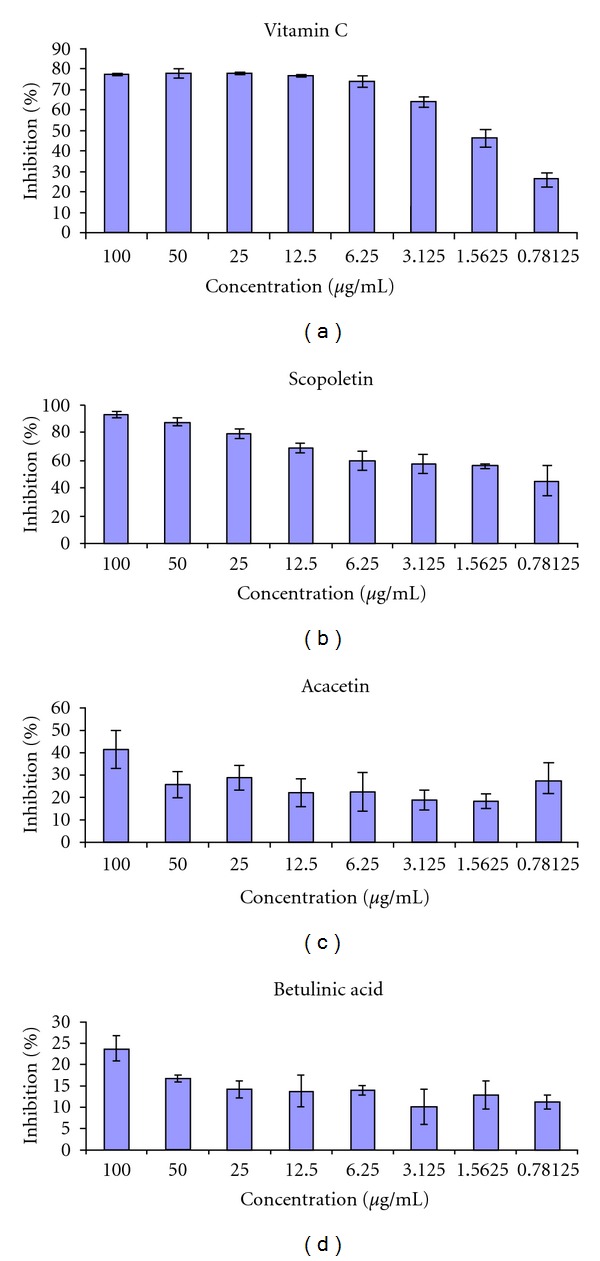
The DPPH inhibitory activities of the isolated compounds and vitamin C.

The antioxidant activity of three selected compounds (scopoletin, acacetin and betulinic acid) revealed that they are effective antioxidant agents (Figure 2). Scopoletin had a strong transformation of the DPPH radical into its reduced form, with an IC_50_ value of 1.24 *μ*g/mL which was in close range to that of vitamin C (1.22 *μ*g/mL). Acacetin and Betulinic acid exhibited a slightly low DPPH scavenging activity with the IC_50_ of 2.39 and 2.42 *μ*g/mL, respectively.

**Figure 3:**
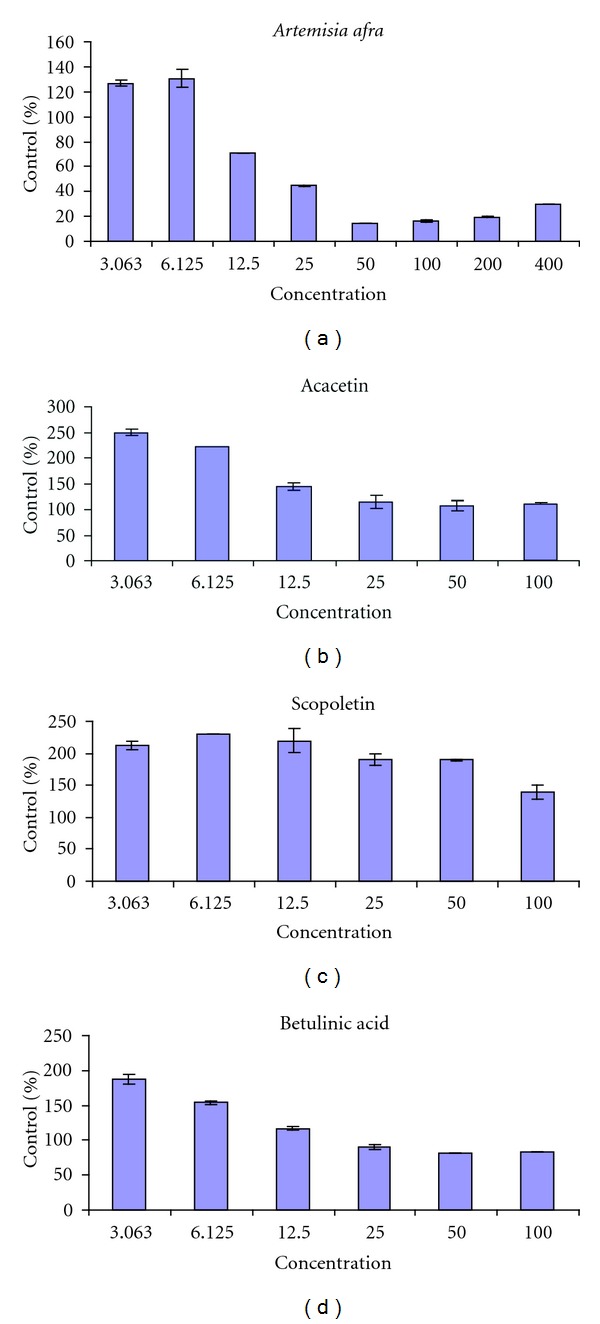
The cytotoxicity effects of *A. afra* extract and three selected compounds on the growth of the McCoy fibroblast cells.

 The cytotoxicity effects of ethanol crude extract of *A. afra* and three selected compound on the growth of Fibroblast cells are shown in Figure 3 and Table 2. The extract as a mixture of different components showed to be nontoxic on lower concentrations of 6.12 and 3.06 *μ*g/mL, with the cell viability of 120%. However, toxic effects were apparent at higher concentration range of 12.50 to 400 *μ*g/mL, with the cell viability of 60 to 20%. Acacetin and betulinic acid also showed a smooth trend of nontoxic effects at lower concentrations and toxic at higher concentrations with IC_50_ values of 35.44 and 30.96 *μ*g/mL respectively. The effect of acacetin on lung cancer (A549) cell proliferation was observed to have a dose-dependent manner with an IC_50_ value of 9.46 *μ*M [32]. Apoptotic activity of betulinic acid against murine melanoma B16 cell line was reported and it was discovered that betulinic acid induces apoptotic effects with an IC_50_ of 22.5 *μ*g/mL [33]. Reports postulates that triterpenes with a carboxyl group at c-28 shows more cytotoxic activity against cancer cell lines [34–36] and induce apoptosis [37]. Unexpectedly, one out of three compounds tested, scopoletin was relatively nontoxic with an IC_50_ value of 132.5 *μ*g/mL. 

## Figures and Tables

**Table 1:** Mean MIC and MMC (mg/mL) results of *Artemisia afra* on oral microorganisms.

Plant extracts	Microorganisms tested											
	MIC (*μ*g/mL)						MBC (*μ*g/mL)					
	Gram +ve		Gram –ve			Yeast	Gram +ve		Gram –ve			Yeast
	A.n	A.i	A.a	P.i	P.g	C.a	A.n	A.i	A.a	P.i	P.g	C.a
*A. afra*	3.1	1.6	25.0	6.3	6.3	6.3	1.6	6.3	>1.0	1.25	25.0	>25.0
**1**	1.0	0.25	>1.0	1.0	1.0	>1.0	1.0	0.5	>1.0	>1.0	>1.0	>1.0
**2**	0.5	0.5	>1.0	1.0	>1.0	>1.0	1.0	0.5	>1.0	1.0	>1.0	>1.0
**3**	1.0	0.25	>1.0	0.5	>1.0	>1.0	>1.0	0.5	>1.0	1.0	>1.0	>1.0
**4**	1.0	>1.0	>1.0	>1.0	>1.0	>1.0	>1.0	>1.0	>1.0	>1.0	>1.0	>1.0
**5**	>1.0	>1.0	>1.0	>1.0	>1.0	>1.0	>1.0	>1.0	>1.0	>1.0	>1.0	>1.0
**6**	0.25	1.0	>1.0	1.0	1.0	>1.0	0.5	1.0	>1.0	>1.0	>1.0	>1.0
Chlorhexidine	1.6	6.3	1.6	6.3	1.6	6.3	1.6	6.3	1.6	6.3	1.6	1.25

A.n: *Actinomyces naeslundii*; A.i:* Actinomyces israelii*; A.a: *Actinobacillus actinomycetemcomitans*; P.i: *Prevotella intermedia*; P.g: *Porphyromonas gingivalis; *C.a:* Candida albicans.*

**Table 2:** The IC_50_ values of crude extract and three selected isolated compounds.

Extract/compounds	IC_50 _(*μ*g/mL)	STD
Betulinic Acid	30.96	±1.95
Acacetin	35.44	±2.14
Scopoletin	132.5	±1.85
*Artemisia afra *	16.95	±1.82
Actinomycin-D	0.003364	±0.00002
